# Aquaporin expression in blood-retinal barrier cells during experimental autoimmune uveitis

**Published:** 2010-04-03

**Authors:** Elie Motulsky, Philippe Koch, Sarah Janssens, Maité Liénart, Anne-Marie Vanbellinghen, Nargis Bolaky, Chi-Chao Chan, Laure Caspers, Maria-Dolores Martin-Martinez, Heping Xu, Christine Delporte, François Willermain

**Affiliations:** 1 Laboratory of Biological Chemistry and Nutrition, Université Libre de Bruxelles, Brussels, Belgium; 2 Department of Ophthalmology, CHU Saint-Pierre and Brugmann, Brussels, Belgium; 3 I.R.I.B.H.M, Campus Erasme, Université Libre de Bruxelles, Brussels, Belgium; 4 Laboratory of Experimental Hormonology, Université Libre de Bruxelles, Brussels, Belgium; 5 National Eye Institute, Bethesda, MD; 6 CMP Laboratory, Brussels, Belgium; 7 Centre for Vision and Vascular Science, Queen’s University Belfast, Belfast, UK

## Abstract

**Purpose:**

Blood-retinal barrier (BRB) breakdown and retinal edema are major complications of autoimmune uveitis and could be related to deregulation of aquaporin (AQP) expression. We have therefore evaluated the expression of AQP1 and AQP4 on BRB cells during experimental autoimmune uveitis (EAU) in mice.

**Methods:**

C57Bl6 mice were immunized with interphotoreceptor retinoid-binding protein (IRBP) peptide 1–16. The disease was graded clinically, and double immunolabeling using glial fibrillary acidic protein (GFAP; a marker of disease activity) and AQP1 or AQP4 antibodies was performed at day 28. AQP1 expression was also investigated in mouse retinal pigment epithelium (RPE) cells (B6-RPE07 cell line) by reverse transcriptase PCR and western blot under basal and tumor necrosis factor α (TNF-α)-stimulated conditions.

**Results:**

In both normal and EAU retina, AQP1 and AQP4 expression were restricted to the photoreceptor layer and to the Müller cells, respectively. Retinal endothelial cells never expressed AQP1. In vasculitis and intraretinal inflammatory infiltrates, decreased AQP1 expression was observed due to the loss of photoreceptors and the characteristic radial labeling of AQP4 was lost. On the other hand, no AQP4 expression was detected in RPE cells. AQP1 was strongly expressed by choroidal endothelial cells, rendering difficult the evaluation of AQP1 expression by RPE cells in vivo. No major differences were found between EAU and controls at this level. Interestingly, B6-RPE07 cells expressed AQP1 in vitro, and TNF-α downregulated AQP1 protein expression in those cells.

**Conclusions:**

Changes in retinal expression of AQP1 and AQP4 during EAU were primarily due to inflammatory lesions, contrasting with major modulation of AQP expression in BRB detected in other models of BRB breakdown. However, our data showed that TNF-α treatment strongly modulates AQP1 expression in B6-RPE07 cells in vitro.

## Introduction

Uveitis is an important cause of blindness worldwide and affects predominantly patients in the working age group [1]. Uveitis can have an infectious etiology or may be autoimmune due to autoreactive lymphocyte activation. Experimental autoimmune uveitis (EAU) is induced in susceptible animals by immunization by retinal proteins or peptides. EAU displays many characteristics of human autoimmune posterior uveitis, with the formation of vitritis, retinal vasculitis, and chorioretinitis [2]. The blood-retinal barrier (BRB) is reported to be severely affected during EAU [3]. This finding is of interest as the major cause of visual loss in uveitis patients is macular edema secondary to BRB disruption [4]. A better understanding of how water flow is regulated during BRB disruption might thus contribute to the development of new therapeutic strategies for the treatment of patients.

BRB is a functional entity that regulates water, solutes, and ions fluxes into the retina. The inner BRB relies on to the isolation of the retina by the tight junctions of retinal vascular endothelial cells [5,6] and its tightness is improved by extensions of Müller cells surrounding retinal blood vessels. The outer BRB relies on the tight junctions of the retinal pigmented epithelial (RPE) cells, which impede any transcellular flow, and on ionic pumps and channels that create a transepithelial osmotic gradient. Under normal conditions water follows this gradient and flows from the subretinal space to the choroidal space through the RPE cells [7]. The exact mechanisms by which water molecules can penetrate the hydrophobic cellular membrane of the RPE cells remain elusive. Aquaporins (AQPs), a family of water-specific membrane-channel proteins, could be good candidates for this function [8]. Indeed, it has been reported that human RPE cells express AQP1 [9]. However, earlier studies reported a lack of expression of AQPs 1, 3, 4, and 5 in human RPE in vivo, suggesting that RPE cells could transport water by AQP-independent mechanisms [10]. On the other hand, AQP4 expression by Müller cells has been consistently described by several groups and is strongest at their perivascular and perisynaptic membrane domains [11–13]. Moreover, Müller cells are thought to be responsible for the dehydratation of the inner retina through a process called “K^+^ siphoning” [6,14]. This process relies on the co-expression of Kir4.1 and AQP4 on Müller cells, which allows water to follow K^+^ from perisynaptic spaces to blood vessels. Interestingly, during endotoxin-induced uveitis, Kir4.1 and AQP4 expression were differentially regulated on Müller cells and the swelling characteristics of these cells were altered by inflammation [15,16]. This finding strongly suggests that the regulation of AQP expression on BRB cells could be critical in the formation of macular edema during uveitis.

The aim of this study was to investigate in vivo the possible modification of the AQP expression pattern on BRB during EAU. In addition, we analyzed the expression of AQP1 in a mouse RPE cell line in vitro and its regulation by tumor necrosis factor α (TNF-α), mimicking the intraocular inflammation (IOI) condition.

## Methods

### Induction of experimental autoimmune uveitis

EAU was induced in C57/Bl6 mice (Iffa Credo, Brussels, Belgium) by subcutaneous 50-µl injections into each thigh with 50 µg (seven mice) or 100 µg (five mice) of peptide corresponding to 1–16 human interphotoreceptor retinoid-binding protein (IRBP; GPTHLFQPSLVLDMA; Sigma-Aldrich, Saint-Louis, MO), emulsified in complete Freund’s adjuvant (supplemented with 2.5 mg/ml mycobacterium tuberculosis; Becton Dickinson, Oxford, UK). The animals received an additional 50-µl intraperitoneal injection of 1 µg of *Bordetella pertussis* toxin on day 0 (Becton Dickinson). Animals were used in agreement with the Association for Research in Vision and Ophthalmology (ARVO) statement for the use of animals in ophthalmic and vision research, and all experimental procedures were approved and performed in compliance with the ethics committees of the Université Libre de Bruxelles, Brussels, Belgium.

### Ocular examination and clinical grading

Twenty-one and 28 days after immunization, mice were submitted to ocular examination following pupil dilatation and fitting of glass coverslips using Vidisic gel (Tramedico, Temse, Belgium). Mouse eyes were positioned in front of a surgical microscope lamp. The onset and the severity of the observable disease were graded according to Xu et al. [17]. Briefly, the onset of the observable disease and its course thereafter were clinically graded on a scale 0–4 depending on the degree of inflammatory lesions of the posterior segment of the eye, considering vitritis, optic neuropathy, vasculitis, retinitis, and retinal folds. As for the characterization of uveitis in human patients (P. Koch, unpublished data), a distinction was made between active an inactive inflammatory lesions in mouse eyes during EAU (P. Koch, unpublished data). Classically, an active phase occurs during uveitis in which inflammatory cells invade the eye. In the case of posterior uveitis during this active phase, fundus examination will demonstrate the presence of active lesions, such as cells in the vitrous, and inflammatory lesions, such as retinal and chorioretinal granulomas, retinal vasculitis (with vessel sheathings, exudates, and hemorrhages), and papillitis. After treatment (or sometimes after a spontaneous natural recovery), these active lesions will give rise to scars or so-called inactive lesions. The distinction between active and inactive lesions can generally be made by fundus examination.

### Tissue preparation and hematoxylin and eosin staining

After 4 weeks of immunization, animals were sacrificed by neck dislocation, and eyes were enucleated. Eyes were immediately fixed in 4% buffered formaldehyde, paraffin-embedded, and sectioned (5-µm thick) at different levels, including in the proximity of the optic nerve. Eye sections were stained with hematoxylin and eosin (Sigma-Aldrich) for histopathological evaluation, and pictures were taken using an Axioplan 2 (Zeiss, Göttingen, Germany).

### Antibodies to aquaporins

Antibodies, resulting from immunization of rabbits using a synthetic peptide corresponding to amino acids 301–318 of mouse AQP4 or to amino acids 245–279 of human AQP1 (this sequence is 100% identical to mouse AQP1), were affinity purified and their specificities verified by western blot and immunohistochemistry (data not shown).

### Immunofluorescence on eye sections

Eye sections were incubated with the primary antibody (rabbit polyclonal affinity-purified anti-AQP1, dilution 1:400; rabbit polyclonal affinity-purified AQP4, dilution 1:200; and a mouse monoclonal to glial fibrillary acidic protein (GFAP; dilution 1:500; Millipore, Billerica, MA), overnight at 4 °C, then incubated with biotinylated antirabbit immunoglobulin G (IgG; 1:200; GE Healthcare, Little Chalfont, UK), streptavidin-cyanin2 (1:300; Jackson Immunoresearch, West Grove, PA), and antimouse IgG coupled to cyanin3 (Jackson Immunoresearch). Cyanin2 and cyanin3 are, respectively, green and red fluorochromes. Cell nuclei were stained with Hoechst (1:5,000; Sigma-Aldrich). Finally, sections were mounted using FluorSave^TM^ reagent (Calbiochem, Gibbstown, NJ). As negative controls sections were incubated either with secondary antibody alone or with anti-AQP antibodies pre-adsorbed with the immunizing peptide in 100-fold excess. Immunofluorescent pictures were taken using an Axiocam MRc 5 fluorescent microscope (Zeiss).

### Cell culture and cell proteins

Mouse RPE cells (B6-RPE07) were grown in Dulbecco’s Modified Eagle Medium (DMEM) containing 10% fetal calf serum, 100 IU/ml streptomycin–penicillin, and routinely passaged twice a week [18]. At confluence, cells were treated for 24 h with 10 ng/ml TNF-α. Cells were lysed in 0.4 ml NP-40 lysis buffer containing 50 mM Tris-HCl (pH 7.5), 150 mM NaCl, 0.5% NP-40, 50 mM NaF, 1 mM sodium orthovanadate, dithiothreitol, and protease inhibitors (Complete EDTA free; Roche, Mannheim, Germany). Cells were then scraped using a rubber scraper, transferred to a tube, frozen in liquid nitrogen, centrifuged for 10 min at 15,000× g, and the supernatant containing the total proteins was collected.

### Reverse transciptase PCR

Total RNA from mouse RPE cells (B6-RPE07 cell line) was extracted using the AURUM^TM^ kit (Bio-Rad, Hercules, CA). The quality of the total RNA was verified using an Experion Automated Electrophoresis System Bioanalyzer (Bio-Rad), and 1 µg total RNA was reverse transcribed using a Revert Aid^TM^ first-strand cDNA synthesis kit (Fermentas, St. Leon-Rot, Germany). The primers used for amplification of mouse cDNA were: sense primer 5′-GCT GTC ATG TAC ATC ATC GCC CAG-3′ nucleotides (nt 472–495) and antisense primer 5′-AGG TCA TTG CGG CCA AGT GAA T-3′ (nt 557–578) for AQP1 (GenBank NM_007472, amplicon 106 bp); and sense primer 5′-TGC ACC ACC AAC TGC TTA GC-3′ (nt 498–517) and antisense primer 5′-GAT GCA GGG ATG ATG TTC T-3′ (nt 655–674) for glyceraldehyde 3-phosphate dehydrogenase (GAPDH; GenBank NM_008084, amplicon 177 bp). PCR reactions were performed in a total volume of 20 µl containing 1 µl of cDNA, 0.5 U GoTaq DNA polymerase (Promega, Madison, WI), 0.2 mM deoxy nucleotides (dNTP), 250 nM of each primer, and 4 µl GoTaq Green buffer using an iCycler System (Bio-Rad). PCR conditions were 94 °C for 3 min, followed by 35 cycles of 30 s at 95 °C, 30 s at 60 °C, and 1 min at 72 °C. PCR products were submitted to electrophoresis on a 1.5% agarose gel.

### Western blot analysis

Total protein preparations were used, and 5% β-mercaptoethanol was added to the sample buffer. The samples were analyzed by sodium dodecyl sulfate PAGE, using 12% polyacrylamide gels. Proteins were transferred to polyvinyldiene difluoride membranes and immunolabeled using the affinity-purified antibody to AQP1 and β-actin at a 1:500 dilution. Bound antibodies were detected using the enhanced chemiluminescence western blotting reagents (GE Healthcare).

## Results

### Aquaporin 1, aquaporin 4, and and glial fibrillar acidic protein expression in normal mouse retina

**Figure 1:**
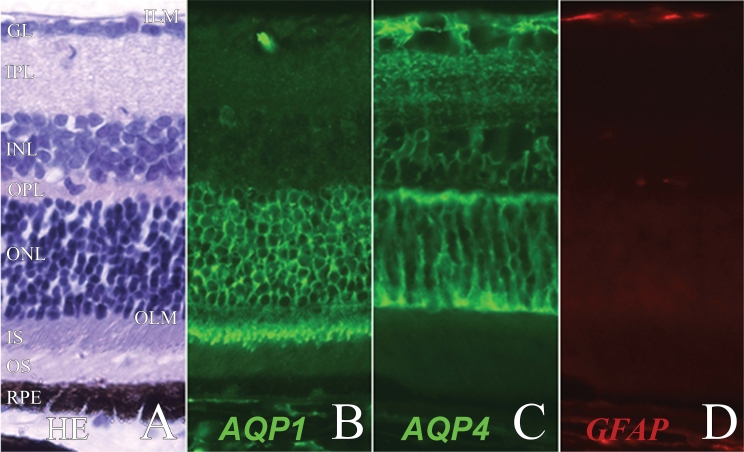
Expression of aquaporin 1, aquaporin 4, and glial fibrillary acidic protein in normal mouse retina. Normal mouse retina was submitted to hematoxylin and eosin staining (**A**), or immunofluorescent staining for aquaporin 1 (AQP1; **B**), aquaporin 4 (AQP4; **C**), and glial fibrillary acidic protein (GFAP; **D**). ILM represents inner limiting membrane; GCL represents ganglion cell layer; IPL represents inner plexiform layer; INL represents inner nuclear layer; OPL represents outer plexiform layer; ONL represents outer nuclear layer; OLM represents outer limiting membrane; IS represents inner segments of the photoreceptors; OS represents outer segments of the photoreceptors; RPE represents retinal pigmented epithelium. Magnification is 200×.

Normal mouse retina has ten well identified histological layers (Figure 1A). AQP1 is expressed in the outer nuclear layer and the inner segment of the photoreceptors (Figure 1B). AQP4 is localized in the Müller cells, from the outer limiting membrane (OLM) to the inner limiting membrane and surrounding the perivascular processes with their endfeet (Figure 1C). Weak GFAP expression is present at the endfeet of some inactivated Müller cells (Figure 1D).

### Disease severity and histopathological lesions

**Figure 2:**
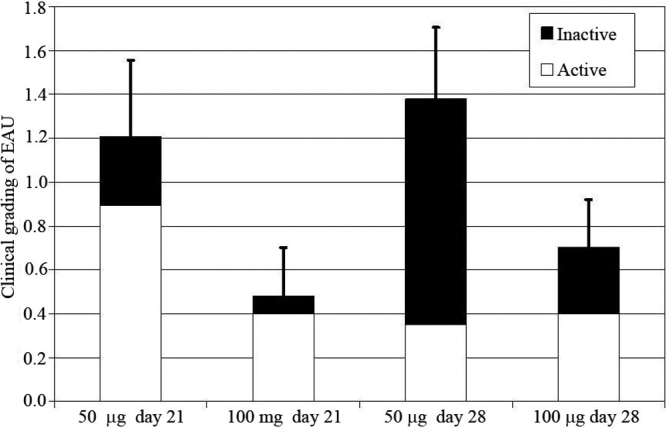
Clinical grading of experimental autoimmune uveitis. Experimental autoimmune uveitis was induced by the subcutaneous injection of 50 µg or 100 µg of peptide corresponding to 1–16 human interphotoreceptor retinoid-binding protein. The clinical grading of active and inactive lesions was performed on a scale of 0–4 at 21 and 28 days post-injection as described in Methods.

EAU was induced in 12 mice. Two groups of mice received an injection of either 50 µg (n=7) or 100 µg (n=5) of IRBP. Three and four weeks post injection, a clinical grading was performed (described above) according to Xu et al. [17]. After 21 days 12 eyes out of 24 (50%) developed an IOI. In the group injected with 50 µg IRBP, eight eyes (57%) had a score between 1.7 and 2.6 on a scale of 4. In the group injected with 100 µg IRBP, four eyes (40%) had an IOI with a clinical grading ranging from 0.4 to 1.9 on a scale of 4. After 28 days, 14 eyes (58%) out of 24 eyes developed an IOI. Among the group (14 eyes) that received 50 µg IRBP, ten eyes (71%) had an IOI: three eyes had a grade between 0.3 and 1.2 on a scale of 4 and seven had a score ranging from 1.8 to 3.2 on a scale of 4. Among the group (ten eyes) that received 100 µg IRBP, four eyes (40%) had a grade between 0.8 and 2.2 on a scale of 4. Figure 2 summarizes the proportion of inactive and active lesions in both groups (50 µg and 100 µg IRBP) at 21 and 28 days post injection.

Histopathological analysis of EAU mouse eyes revealed the presence of focal lesions, such as intraretinal inflammatory infiltrate and retinal architectural disorganization with some destruction of the RPE cell monolayer, vitritis, retinal folds in the layers extending from the outer plexiform layer to the RPE, and vasculitis characterized by various inflammatory cells surrounding a congested and dilated retinal vessel (data not shown).

### Aquaporin 1, aquaporin 4, and glial fibrillary acidic protein expressions during experimental autoimmune uveitis

**Figure 3:**
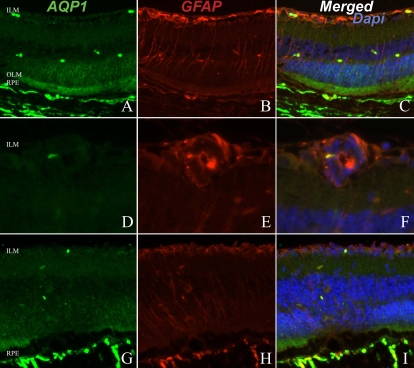
Retinal expression of aquaporin 1 and glial fibrillary acidic protein during experimental autoimmune uveitis. The images illustrate retina without specific lesions (**A, B**, and **C**), with vasculitis (**D, E**, and **F**) or with intraretinal inflammatory infiltrate (**G, H**, and **I**). Retina was submitted to immunofluorescent staining for aquaporin 1 (AQP1; in green; **A, D,** and **G**), or to glial fibrillary acidic protein (GFAP; in red; **B, E,** and **H**). Cell nuclei were stained with DAPI (blue). **C**, **F,** and **I** correspond to merged images. ILM represents inner limiting membrane; OLM represents outer limiting membrane; and RPE represents retinal pigmented epithelium. Magnification is 60×.

**Figure 4:**
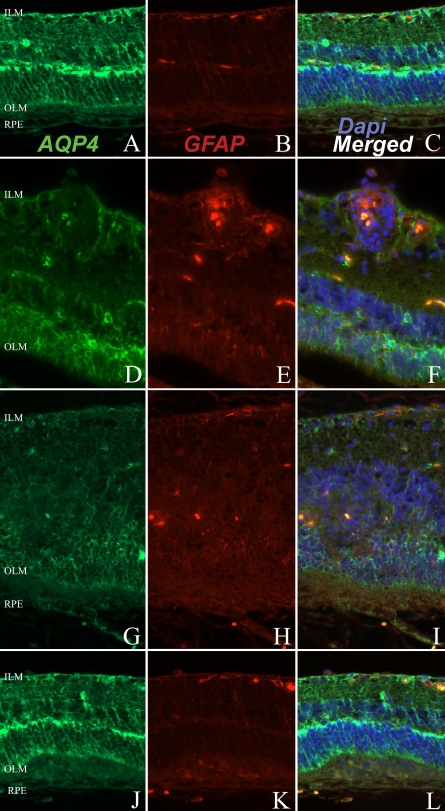
Retinal expression of aquaporin 4 and glial fibrillary acidic protein during experimental autoimmune uveitis. The images illustrate retina without specific lesions (**A, B,** and **C**), with vasculitis (**D, E**, and **F**) or with intraretinal inflammatory infiltrate (**G, H, I, J, K**, and **L**). Retina was submitted to immunofluorescent staining for aquaporin 4 (AQP4; in green; **A, D, G**, and **J**), or to glial fibrillary acidic protein (GFAP; in red; **B, E, H,** and **K**). Cell nuclei were stained with DAPI (in blue). **C**, **F**, **I,** and **L** correspond to merged images. ILM represents inner limiting membrane; OLM represents outer limiting membrane; RPE represents retinal pigmented epithelium. Magnification is 60×.

To investigate the possible role of AQPs in the development of inflammatory retinal edema, the expression of AQP1 and AQP4 was analyzed during EAU. Changes in GFAP expression, described during retinal inflammation and used as a marker for disease activity [19,20], were also investigated in our model of EAU. In control animals GFAP was mainly located in the ganglion cell layer (Figure 1D), while in eyes developing intraocular inflammation GFAP expression is located in activated Müller cells and retinal glial cells, extending from the inner limiting membrane to the OLM (Figure 3B and Figure 4B). This pattern of GFAP expression was consequently used as an indicator of intraocular inflammation. In normal (Figure 1B) and EAU (Figure 3A) retinas, AQP1 expression was restricted to the photoreceptor layer with an intense staining in the outer nuclear layer and the photoreceptor inner segments. In the inner retina, some red blood cells inside the retinal capillaries were also positive for AQP1 (Figure 1B, Figure 3A). Strikingly, retinal endothelial cells did not express AQP1 in normal or EAU retina, even on vasculitis lesions (Figure 1B, Figure 3A,D). In focal retinal inflammatory lesion, AQP1 intensity was lower due to the loss of photoreceptors (Figure 3G).

As in normal retina, AQP4 was expressed in Müller cells in EAU retina (Figure 4A). The staining was still intense in the ganglion cell layer, the outer plexiform layer and the OLM (Figure 4A). However, in vasculitis (Figure 4D) and in retinal inflammatory lesions (Figure 4G), the characteristic radial labeling of AQP4 observed in controls was lost.

**Figure 5:**
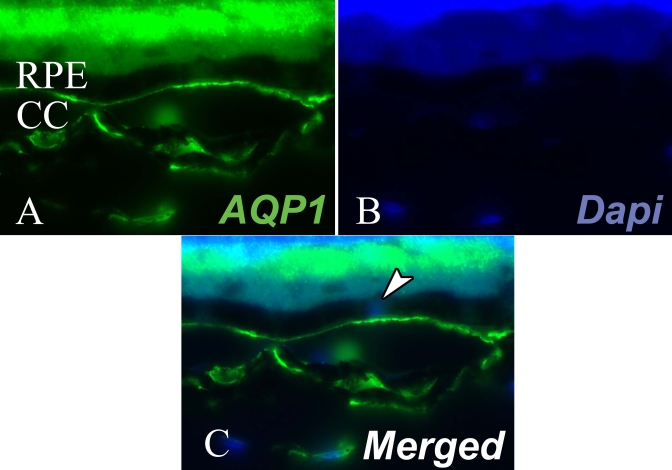
Subretinal expression of aquaporin 1 during experimental autoimmune uveitis. Retina was submitted to immunofluorescent staining for aquaporin 1 (green; **A**). Cell nuclei were stained with DAPI (blue, **B**). **C** corresponds to merge image. The arrow shows the cell nuclei of the RPE cells monolayer. RPE represents retinal pigmented epithelium and CC represents choriocapillaris. Magnification is 400×.

We carefully examined AQP1 and AQP4 expression in RPE cells, corresponding to the external BRB. In normal and EAU retina, AQP4 immunofluorescent staining was totally absent from the OLM of the RPE cell monolayer, suggesting that AQP4 is not expressed in RPE cells (data not shown). Choroidal endothelial cells strongly expressed AQP1, and no differences were found between EAU and controls. The intensity of the AQP1 staining by endothelial cells and their proximity with RPE cells render the evaluation of AQP1 expression by RPE cells in vivo difficult (Figure 5).

### Expression of aquaporin 1 and aquaporin 4 by mouse retinal pigmented epithelial cells in vitro (B6-RPE07 cell line)

**Figure 6:**
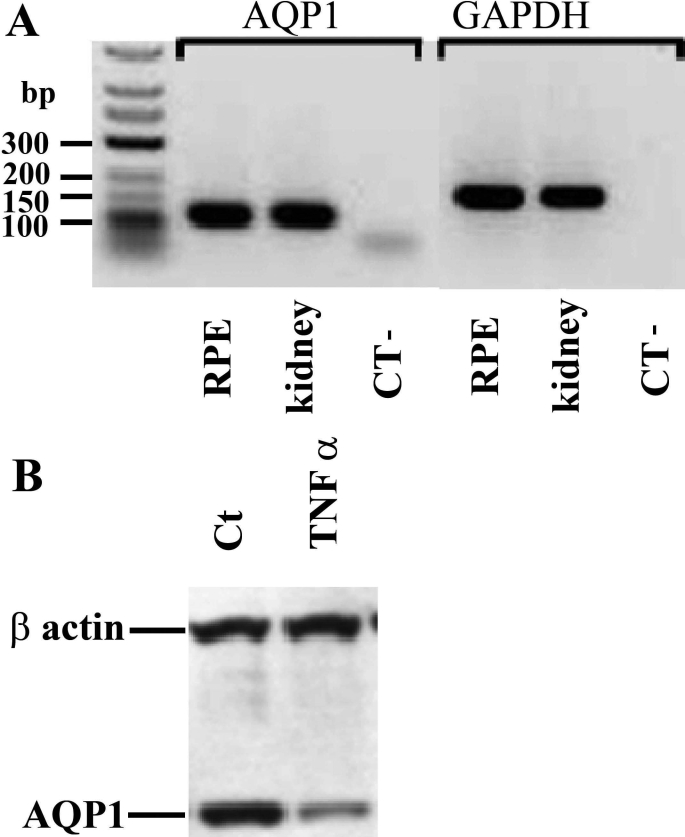
Expression of aquaporin 1 in mouse B6-RPE07 cells. **A**: The presence of aquaporin 1 (AQP1) and glyceraldehyde 3-phosphate dehydrogenase (GAPDH) mRNA expression was detected by reverse-transcriptase PCR using B6-RPE07 cell cDNA (RPE), kidney cDNA (kidney) used as positive control, or nontarget control (CT-). Molecular weight ladders are shown. **B**: Aquaporin 1 (AQP1) and β-actin (ßactin) protein expressions were detected by western blot in B6-RPE07 cells treated without (Ct) or with 10 ng/ml tumor necrosis factor (TNFα). The blot is representative of three experiments performed using distinct cell preparations.

Due to the critical role of RPE cells in the regulation of retinal water content and their implication in the development of uveitis, AQP1 expression was further analyzed in the recently described clone B6-RPE07 which has a morphology, phenotype, and function similar to those of mouse RPE cells in vivo [18]. Both reverse transcriptase PCR (Figure 6A) and western blot (Figure 6B) revealed the expression of AQP1 in B6-RPE07 cells. Furthermore, TNF-α treatment downregulatedAQP1 expression in those cells (Figure 6B).

## Discussion

BRB cells regulate water movement in the retina. In the inner part of the BRB, water follows the osmotic-driven potassium flow and is extruded through AQP4 channels expressed on Müller cells. In the subretinal space, water also follows the direction of the osmotic gradient and flows to the choriocapillaries through the tight RPE cell monolayer. AQP expression on RPE cells is still controversial [21]. Furthermore, the mechanisms responsible for water accumulation in the retina during macular edema are still poorly understood. Nevertheless, it is tempting to postulate that modulation of AQP expression is involved in macular edema formation, as shown in brain edema as well [22,23].

Concerning the expression of AQPs in retina, our data confirm the expression of AQP4 in Müller cells [11] and AQP1 in photoreceptors [24]. Furthermore, neither AQP1 nor AQP4 expression was found on retinal vascular endothelial cells. However, AQP1 expression was strongly expressed on choroidal vascular endothelial cells. Interestingly, we additionally found that AQP1 and AQP4 expression patterns moderately altered during EAU compared to control probably because of retinal restructuring during EAU. These latter data are consistent with the slightly reduced expression of AQP4 observed during endotoxin-induced uveitis [15]. In contrast, modulation of retinal AQP1 and AQP4 expressions was observed during experimental diabetes [25]; AQP1 expression was induced on glial cells and in the innermost retinal layers [25]. However, retinal vascular endothelial cells did not express AQP1 or AQP4 [25]. The absence of AQP1 expression in retinal vascular endothelial cells is consistent with our data and those from Verkman’s group [21]. Altogether, these data emphasize the role of Müller cells in retinal water transport and suggest that AQPs might be differentially regulated during uveitis and diabetes.

No AQP4 expression was found in the RPE cells monolayer under normal conditions or in EAU. Despite the important role assigned to RPE cells in BRB water flow regulation, the possible expression of the AQPs remains controversial [21]. Indeed, Hamann et al. [10,26] did not find AQP1 expression in rat RPE cells but described a strong AQP1 immunolabeling in the posterior pigmented epithelium of the iris. In contrast, human RPE cells were reported to express AQP1 in situ, and RPE cells in primary culture continued to express AQP1, although the expression level was significantly lower [9]. In the present work, we found that the intense expression of AQP1 by choroidal endothelial cells renders the analysis of AQP1 expression by RPE cells in vivo difficult. Therefore, the expression of AQPs in RPE cells was analyzed in vitro. Our data showed that mouse RPE cells in vitro express AQP1 and that AQP1 expression is downregulated following TNF-α stimulation.

In conclusion, in contrast to other models of BRB breakdown, major modulation of AQP1 and AQP4 retinal expression were not found during EAU. Our data also indicate that AQP1 is indeed expressed in RPE cells and is downregulated following TNF-α treatment. Furthermore, the latter observation supports in vivo expression of AQP1.
